# Cold-inducible proteins CIRP and RBM3, a unique couple with activities far beyond the cold

**DOI:** 10.1007/s00018-016-2253-7

**Published:** 2016-05-04

**Authors:** Xinzhou Zhu, Christoph Bührer, Sven Wellmann

**Affiliations:** 1 University Children’s Hospital Basel (UKBB), Spitalstrasse 33, 4056 Basel, Switzerland; 2 Department of Neonatology, Charité University Medical Center, Berlin, Germany; 3 University of Basel, Basel, Switzerland

**Keywords:** Transcription, Translation, hnRNP, MicroRNA, Neuroscience, Apoptosis, Stress granule

## Abstract

Cold-inducible RNA-binding protein (CIRP) and RNA-binding motif protein 3 (RBM3) are two evolutionarily conserved RNA-binding proteins that are transcriptionally upregulated in response to low temperature. Featuring an RNA-recognition motif (RRM) and an arginine–glycine-rich (RGG) domain, these proteins display many similarities and specific disparities in the regulation of numerous molecular and cellular events. The resistance to serum withdrawal, endoplasmic reticulum stress, or other harsh conditions conferred by RBM3 has led to its reputation as a survival gene. Once CIRP protein is released from cells, it appears to bolster inflammation, contributing to poor prognosis in septic patients. A variety of human tumor specimens have been analyzed for CIRP and RBM3 expression. Surprisingly, RBM3 expression was primarily found to be positively associated with the survival of chemotherapy-treated patients, while CIRP expression was inversely linked to patient survival. In this comprehensive review, we summarize the evolutionary conservation of CIRP and RBM3 across species as well as their molecular interactions, cellular functions, and roles in diverse physiological and pathological processes, including circadian rhythm, inflammation, neural plasticity, stem cell properties, and cancer development.

## Introduction

Decreased body temperature is a key feature of seasonal hibernation, which is an entrained state of slowed metabolism that widely exists in amphibians and mammals to endure food austerity [1–3]. As a direct cellular consequence of decreased body temperature, global protein synthesis is repressed, thereby switching the cellular program from cell growth to cell preservation. In contrast to the general decrease in protein synthesis, the production of a small group of proteins, including cold-inducible RNA-binding protein (CIRP, alternative abbreviation: CIRBP; synonymous: heterogeneous ribonucleoprotein A18, hnRNP A18) and RNA-binding motif protein 3 (RBM3), increases in hibernating animals [4, 5].

In clinical practice, therapeutic hypothermia (32–34 °C) has been proved a potent tool to alleviate neurological deficits in infants with hypoxic-ischemic encephalopathy [6], and in adults with acute brain injuries [7]. Whereas much deeper hypothermia is used during cardiac and transplant surgery [8], CIRP and RBM3 protein syntheses peak in a range of mild to moderate temperatures (32–34 °C) [9]. Because clinical hypothermia is associated with various life-threatening side effects [10], CIRP and RBM3 are promising research candidates for new therapies.

Apart from their functions under hypothermia, various studies have indicated that CIRP and RBM3 also have important functions in cell protection under general endogenous and environmental stresses at normal temperatures [11]. Here, we provide a comprehensive and systematic overview of biological functions mediated by CIRP and RBM3, within and outside the context of hypothermia by systematically considering almost all papers published on CIRP and RBM3 thus far.

## Evolution and protein structure

Whereas the gene coding for human CIRP is localized on chromosome 19p13.3 [12], the gene coding for human RBM3, the homolog of CIRP, has been mapped to the short arm of the X-chromosome at Xp11.23 [13].

**Fig. 1:**
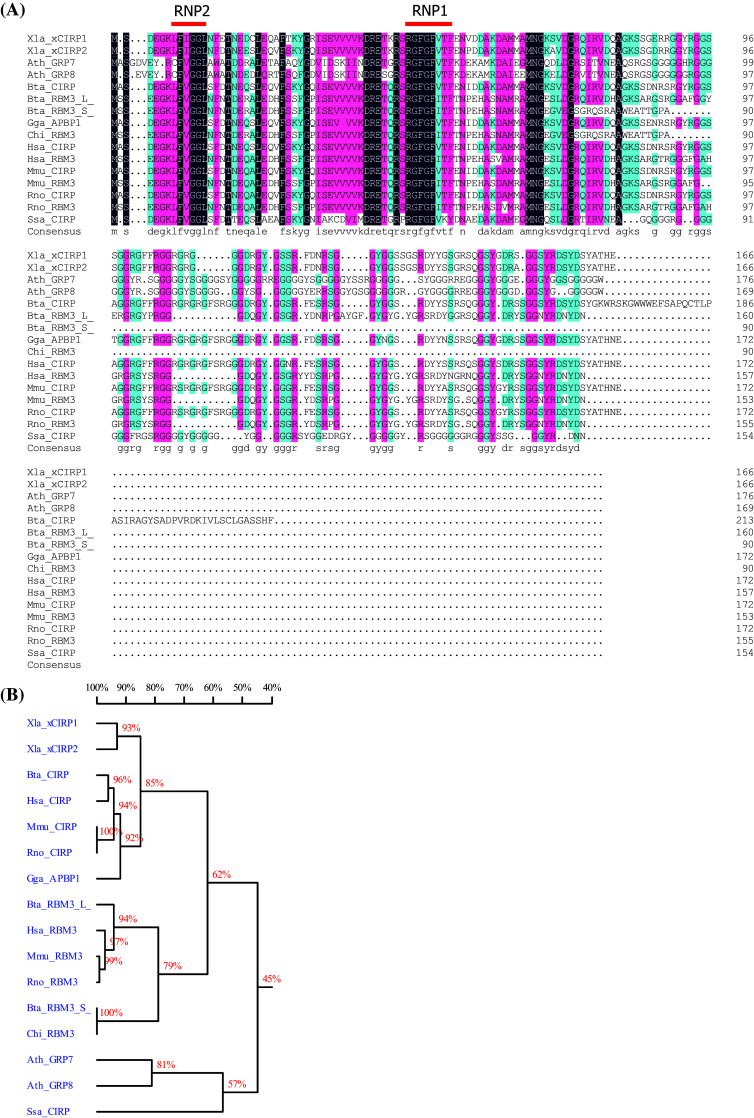
Protein alignment (**a**) and homology tree (**b**) of CIRP, RBM3, and their plant homologues in different species. *Xla* *Xenopus laevis*, *Ath* *Arabidopsis thaliana*, *Bta* *Bos Taurus*, *Gga* *Gallus gallus*, *Chi* *Capra hircus*, *Hsa* *Homo sapiens*, *Mmu* *Mus musculus*, *Rno* *Rattus norvegicus*, *SSa* *Salmo salar*, *L* long full-length RBM3, *S* short truncated RBM3

Both CIRP and RBM3 belong to a group of stress-responsive proteins that share high amino acid sequence similarity in the N-terminal RNA-binding domain (Fig. 1; Table 1). They both own one conserved RNA-recognition motif (RRM), containing two ribonucleoprotein domains (RNPs), RNP1 and RNP2, which are located at the N-terminal protein end. The consensus sequences of RNP1 and RNP2 are (K/R)G(F/Y)(G/A)FVX(FY) and (L/I)(F/Y)(V/I)(G/K)(G/N)L, respectively [14]. Notably, both RNP1 and RNP2 from CIRP and RBM3 show moderate sequence and function similarity to parts of cold shock proteins (CSPs), with the evolutionary conserved sequences (K/S) G(F/K/Y)G(F/L)IXX and (L/I/V)(F/Q)(V/A/L)HX(STR), respectively [15]. Prokaryotic CSPs exert important functions in response to drastic drops in temperature, e.g., from 37 to 10 °C [14, 15], indicating common features as well as differences between CIRP/RBM3 and CSPs.

**Table 1:** Proteins used for alignment in Fig. 1a

	Species	Name abbreviation	Reference ID in UniProtKB	Full name
Plant	*Arabidopsis thaliana*	Ath_GRP7	Q03250	Glycine-rich RNA-binding protein 7
	*Arabidopsis thaliana*	Ath_GRP8	Q03251	Glycine-rich RNA-binding protein 8
Fish	*Salmo salar*	Ssa_CIRP	B5DGC5	Cold-inducible RNA-binding protein
Amphibian	*Xenopus laevis*	Xla_xCIRP1	O93235	Cold-inducible RNA-binding protein A
	*Xenopus laevis*	Xla_xCIRP2	Q9DED4	Cold-inducible RNA-binding protein B
Bird	*Gallus gallus*	Gga_CIRP	Q45KQ2	Aggrecan promoter binding protein (CIRP homologue)
Mammal	*Bos Taurus*	Bta_CIRP	Q3SZN4	Cold inducible RNA binding protein
	*Bos Taurus*	Bta_RBM3_L	F6RBQ9	Uncharacterized protein
	*Bos Taurus*	Bta_RBM3_S	Q3ZBA4	RNA binding motif (RNP1, RRM) protein 3
	*Capra hircus*	Chi_RBM3	W8E7I1	RNA-binding protein 3
	*Mus musculus*	Mmu_CIRP	P60824	Cold-inducible RNA-binding protein
	*Mus musculus*	Mmu_RBM3	O89086	RNA-binding protein 3
	*Rattus norvegicus*	Rno_CIRP	P60825	Cold-inducible RNA-binding protein
	*Rattus norvegicus*	Rno_RBM3	Q925G0	RNA-binding protein 3
	*Homo sapiens*	Hsa_CIRP	Q14011	Cold-inducible RNA-binding protein
	*Homo sapiens*	Hsa_RBM3	P98179	RNA-binding protein 3

The C-terminal part of both CIRP and RBM3 contains a less conserved arginine–glycine-rich (RGG) domain, for which reason CIRP and RBM3 belong to the large family of glycine-rich proteins (GRP). In addition, because they are featured with an RRM, they belong to the subfamily Class IVa of GRPs [14, 16]. The evolution of this Class IVa GRP subfamily is highly conserved across vertebrates and higher plants [14] (Fig. 1) with respect to their primary amino acid sequences as well as to their protein functions. For example, in *Arabidopsis*, the CIRP/RBM3 homologue AtGRP7 is indispensable in cold adaption and drought/osmotic stress responses [17–19]. AtGRP7 also regulates a number of post-transcriptional and translational events [20–23], functions as a circadian oscillator [24], and is involved in pathogen defense [25, 26]. In poikilothermic animals, such as fish, CIRP homologues are also elevated upon environmental osmotic or severe cold stresses (8 °C) but may not change at normal ambient temperatures (20–25 °C) [27–29]. As detailed in the following paragraph, the well-studied amphibian and mammalian CIRP and RBM3 possess biological functions highly similar to AtGRP7 and fish CIRP, implying their preservation of biological activities.

## Temporal and spatial distribution

Both CIRP and RBM3 are key factors during early development. In amphibians, which are widely used as models for developmental studies, *Xenopus laevis* homologue xCIRP-1 is transiently expressed in the developing kidney and brain [30] and is required for embryonic kidney formation [31]. The expression of CIRP homologue AxRBP in the Mexican axolotl starts at gastrula stage day 10–12, peaks at neurula stage day 15 particularly in the neural plate and neural fold, and declines afterward to low levels when hatching [32]. In mammals, RBM3 level peaks during the early postnatal period and then decreases to very low levels in youth and adulthood in most regions of the brain, except for areas where proliferation remains active, such as the subventricular zone (SVZ) and the hippocampal subgranular zone (SGZ) [33, 34], indicating a pivotal role of RBM3 in the maintenance of stemness and proliferation in neural stem/progenitor cells. Regardless to this dynamic temporal expression of CIRP and RBM3 with high abundance in early developmental stages and low in mature organisms, many mature cells maintain their ability to overexpress CIRP and RBM3 in response to stressful conditions, such as cold, see below for details.

The spatial distribution of CIRP and RBM3 in major organs varies between species. In human, RBM3 expression is low or absent in thyroid and heart, whereas CIRP is abundant in these organs [35, 36]. In hibernating animals, RBM3 is upregulated in muscle, liver, and heart tissues of black bears [37, 38], as well as in brain, heart, and liver tissues of squirrels at late torpor [4]. In contrast, CIRP fails to be stimulated in muscle and liver tissues in rats with chronic intermittent cold exposure, but is induced in brain and heart [39]. Within the same tissue, their spatial patterns can be cell type-specific: in mammalian testis, CIRP is predominantly in germ cells [40], whereas RBM3 is mainly in Sertoli cells [41].

At subcellular level, the spatial distribution of CIRP and RBM3 is expected to be mainly in the nucleus, because both proteins are featured with an RGG domain which is a nuclear localization signal and associated with nucleocytoplasmic shuttling. In fact, CIRP and RBM3 are predominantly found in the nucleus [42], where they regulate gene transcription or bind to mRNA for post-transcriptional regulation. In addition, under physiological or stressful conditions, CIRP and RBM3 shuttle between nucleus and cytoplasm [43]. However, there are at least three important exceptions, indicating that the subcellular localization of RBM3 and CIRP is subjected to developmental stage and cell type. First, during the first week after birth, RBM3 resides in the nucleus, then shifts during the second postnatal week toward a more cytoplasmic localization. In sections of adult brain, RBM3 is in general very weekly expressed, and the balance of RBM3 subcellular localization also appears to be highly dependent on cell type [33]. Second, frog xCIRP2 has been found to serve as a major cytoplasmic protein in oocytes [44]. Third, in contrast to strong expression of CIRP in the nucleus of spermatocytes in both mice and humans, round spermatids at stages I–III of mice show CIRP expression in cytoplasm but not in the nucleus, suggesting an additional function of CIRP in the cytoplasm of haploid cells [40].

Interestingly, a nucleocytoplasmic shuttling signal (RG4) has been identified within the RGG domain of frog xCIRP2, which promotes accumulation of xCIRP2 in the cytoplasm once methylated by arginine methyltransferase xPRMT1 [45]. In mammals, methylation of arginine residues in the RGG domain of CIRP due to cytoplasmic stress and endoplasmic reticulum (ER) stress causes CIRP accumulation in cytoplasmic stress granules independent of the major mediator of stress granule formation TIA-1 [46]. For RBM3, a distinct pool of RBM3 proteins can shuttle to the ER upon ER stress where RBM3 regulates the activity of ER membrane protein PERK. The majority of RBM3 proteins, however, remain in the nucleus [47]. The absence of a single arginine residue in the RGG domain of RBM3 promotes the localization of RBM3 in dendrites of neurons rather than in nuclei [48]. Collectively, these studies reveal a critical role of the arginine residue in the RGG domain of CIRP and RBM3 in nucleocytoplasmic shuttling.

## Stress-regulated expression

*Hypothermia and hyperthermia*: *CIRP* and *RBM3* are stress-responsive genes, and their expression is induced by a variety of stressful conditions, including cold stress, which is the first identified condition that increases CIRP and RBM3 expression [35, 49]. In the very early beginnings of CIRP and RBM3 research, both proteins were discovered with high expression in mammalian testis, an organ located outside the body to maintain temperatures slightly less than core body temperature to ensure efficient spermatogenesis [41]. Expression of CIRP and RBM3 decreases under experimental heat stress or cryptorchidism, with a more rapid response of CIRP (6 h) to hypothermia than RBM3 (12 h) [40, 41].

In mammalian cells, the expression levels of both CIRP and RBM3 reach their peaks upon mild to moderate hypothermia (28–34 °C) and drop significantly upon deep hypothermia (15–25 °C) [9, 42, 49]. In contrast, hyperthermia (39–42 °C) causes substantial decreases in CIRP and RBM3 in cultured cells in vitro [35, 49], which is consistent with their change under pathological and experimental conditions in vivo [40, 41]. Notably, RBM3 induction is extremely sensitive to temperature change at least in neural cells, even a 1 °C drop from 37 to 36 °C is sufficient [50]. These facts indicate that CIRP and RBM3 respond to temperature change within a small range in a subtle manner.

In cultured tissues or cells, CIRP also increases more rapidly than RBM3 upon moderate hypothermia at early stage, but declines faster during subsequent rewarming process, indicating a faster dynamic change of CIRP in cold response [9, 51]. When cells are exposed to hypothermia, CIRP is activated within 3 h and reaches maximal expression at 12 h, but drops by 50 % within 8 h during rewarming. In contrast, RBM3 is induced after 3 h and peaks at around 24 h, and its expression level remains unchanged until 8 h after rewarming [11, 35, 51]. However, the dose–response kinetics of CIRP and RBM3 are dependent on the biological systems studied [9, 35, 42, 51].

*Hypoxia*: Under natural circumstances and when breathing air containing 21 % oxygen at sea level, the oxygen concentrations in the different tissues of the body are considerably heterogeneous [52]. Reduced oxygen tension (hypoxia) compared with physiological tension occurs during diverse acute and chronic injuries or diseases, including cancer [53]. Experimentally, both mild (8 %) and severe (1 %) hypoxia can induce CIRP and RBM3 expression to a comparable level by a mechanism that involves neither hypoxia-inducible factor (HIF) nor mitochondria in vitro [54]. In contrast, severe hypoxia mimicking ischemia in an in vitro model applying cultured neural stem cells (NSCs) suppresses CIRP expression in parallel with cell cycle arrest [55]. Since hydrogen peroxide treatment can inhibit the hypothermia-induced expression of CIRP [56, 57] and a mild level of reactive oxygen species (ROS) is beneficial, whereas a high level is toxic for NSC proliferation, it has been hypothesized that mild hypoxia resulting in mild elevation of ROS increases CIRP expression, whereas severe hypoxia/ischemia inducing an overload of ROS suppresses CIRP expression [55]. Exposing pregnant mice in late gestation to severe systemic hypoxia causes overexpression of HIF-dependent genes and downregulation of RBM3 expression in the placenta and developing brain [58]. In summary, oxygen-regulated expression of CIRP and RBM3 is dose-dependent and subjected to cell vulnerability, and other factors involved in development or pathological changes, such as hypoxic-ischemia, carcinogenesis, and inflammation.

*Radiation*: Independent of the first characterization of CIRP from Nishiyama et al. in 1997, Sheikh et al. identified the UV light-induced heterogeneous nuclear ribonucleoprotein A18 (hnRNP A18), which was soon proved to be a CIRP homologue in hamster playing a role in DNA damage repair [59]. Similarly, ionizing radiation can stimulate a number of hnRNPs, including CIRP, which promotes the repair of radiation-induced DNA damage [60]. Furthermore, spaceflight increases CIRP and RBM3 expression [61, 62], which may result from irradiation in space.

*Miscellaneous*: Toxins and drugs can also promote CIRP and RBM3 induction. For instance, the neurotoxin domoic acid elevates *CIRP* and *RBM3* mRNA expression in mouse brain [63]. In fish, *CIRP* mRNA expression is upregulated upon lipopolysaccharide (LPS) treatment [64]. In addition, as shown in mouse leukemic cell line, RBM3 forms together with other RNA-binding proteins an RNA–protein complex with the first 60 nucleotides of the 3′-UTR of *cyclooxygenase*-*2* (*COX*-*2*) mRNA [65]. Growth factors, such as insulin-like growth factor-1 (IGF-1) and fibroblast growth factor 21 (FGF21), can induce CIRP and RBM3 expressions, respectively [50, 66]. Melatonin, a well-studied hormone with both endogenous and exogenous sources, may augment the induction of RBM3 upon mild hypothermia in young neurons but not in mature neurons [50]. Of note, very recently, RBM3 has been shown to be suppressed by metformin and the AMP analog AICAR, probably via the inference of cell metabolism and the activation of AMPK [67]. These observations indicate that stress is not always an inducer, but can be an inhibitor of cold-inducible proteins as well.

## Regulation and functions of CIRP

### Molecular regulation of CIRP

The precise mechanism by which hypothermia and other stresses modulate the transcription and translation of cold-inducible proteins is poorly understood, although several models have been suggested involving various regulatory levels. At the transcriptional level, a core promoter and an alternative promoter have been identified in the mouse *CIRP* gene, and both promoters are activated upon mild hypothermia [68]. Furthermore, alternative splicing is one important route in response to cold stress. Hamsters, which are non-hibernating animals, express a long *CIRP* transcript in their hearts. This transcript has an extra insert containing a stop codon inside the open reading frame (ORF), which probably leads to a truncated translational product and aberrant function. In contrast, hibernating animals predominantly express the short isoform with a complete ORF. Artificial hypothermia can partially promote a shift from the long isoform to the short functional isoform [5]. In mouse fibroblasts, the 5′-UTR and full-length ORF of *CIRP* are present in two transcripts that are generated under hypothermic conditions, whereas the isoform under euthermic conditions lacks the 5′-UTR and the code for the initial methionine [69]. Cold stress upregulates the level and stability but not translational efficiency of the longer *CIRP* transcript, which contains a putative internal ribosome entry site (IRES) in the 5′-UTR [69]. In addition, transcription factors may contribute to the modulation of cold-inducible gene transcription. At 32 °C, a greater number of the transcription factor Sp1 are recruited to the mild-cold responsive element (MCRE) in the 5′-flanking region of the *CIRP* gene than at 37 °C, leading to increased CIRP expression [70]. Overall, CIRP expression levels are altered in response to stress by versatile mechanisms, suggesting a wide range of adaptation to various external and internal challenges.

### Molecular and cellular activities of CIRP

#### Regulation of post-transcriptional and translational events

As other RNA-binding proteins, CIRP has the capacity to bind RNAs, and to modulate them at the post-transcriptional level [71, 72]. In general, such post-transcriptional interactions by RNA-binding proteins involve binding to the target regions within the 3′-UTR, which spans the nucleotide sequence between the stop codon and poly(A) tail [73]. Upon UV irradiation, CIRP binds to the 3′-UTR of two stress-responsive transcripts, *replication protein A* (*RPA*) and *thioredoxin* (*TRX*), thereby stabilizing the bound mRNA and promoting their translation [74, 75]. Both CIRP RRM domain and RGG domain are required for the maximal binding activity to *TRX* mRNA [75]. These domains bridge 5′- and 3′-UTRs of the *TRX* transcript via eIF4G, a key component of the translational machinery to enhance TRX translation [75]. A CIRP-binding motif found in *RPA* and *TRX* 3′-UTRs also exists in the 3′-UTR of *ataxia telangiectasia mutated*- and *Rad3*-*related* (*ATR*) mRNA, a key regulator of the DNA damage response [76]. Thus, CIRP-mediated repair of UV-induced DNA damage involves at least partially ATR [76]. In rat ventricular myocytes, *Cirp* ablation upregulates the protein levels of potassium channels by post-transcriptional modulation of their α-subunits without changing their transcriptional activity [77].

In addition to the 3′-UTR, the poly(A) tail is an important regulatory element for CIRP-mediated post-transcriptional modulation. Actually, CIRP is enriched in poly(A) sites and controls alternative polyadenylation of a variety of genes, including circadian genes [78]. Moreover, in the regulation of *TRX* mRNA, the poly(A) tail can strengthen the binding of CIRP to the *TRX* 3′-UTR and enhance its stability [74]. Furthermore, CIRP putatively associates with the spliceosome [79].

Another RNA-binding protein, human antigen R (HuR), which is known to bind to AU-rich elements of 3′-UTR [80], can strengthen CIRP-mediated regulation. In African-clawed frog, the CIRP homolog xCIRP2 interacts with ElrA (an HuR homologue), and both xCIRP2 and ElrA stabilize mRNA in a cooperative manner [81]. Co-regulation of *cyclin E1* mRNA stability by HuR and CIRP has also been discovered in mammalian cancer cells [82].

The function of CIRP in protein translation is unclear. CIRP can associate with ribosomes [44]. The RGG domain of CIRP tethers 3′-UTR and suppresses translation [46, 83]. In addition, there is some evidence to show that CIRP may inhibit gene transcription and translation by targeting regulatory elements inside genes. For example, APBP-1, the chicken homologue of CIRP, binds to a *cis*-element of the aggrecan gene and represses its expression [84]. In contrast, overexpression of CIRP at 37 °C in an engineered CHO cell line improves the production of recombinant interferon gamma protein [85].

### Signaling pathways

As a regulatory protein, CIRP is involved in complex signaling pathways relating to diverse cellular physiological processes, such as cell growth, senescence, and apoptosis.

*Stemness*: The Wnt/β-catenin pathway is one of the main pathways controlling self-renewal of stem and progenitor cells [86]. The frog xCIRP is a target of xTcf-3, a key mediator in the Wnt/β-catenin pathway [87]. The endogenous inhibitor of β-catenin, GSK-3β kinase, upregulates *CIRP* transcription, phosphorylates CIRP protein and promotes its cytosolic translocation [75, 76, 88]. Furthermore, CIRP maintains the expression of adhesion molecules, including β-catenin, and is required for embryonic cell movement during development [89].

*Cell cycle*: Hypothermia is known to slow cell proliferation and to cause cell cycle arrest. In contrast to an early study, which has shown an inhibitory role of CIRP in cell growth upon hypothermia [49], a more recent series of mechanistic investigations have revealed that CIRP positively modulates the cell cycle at different stages. CIRP interacts with HuR and upregulates its expression. In cooperation with CIRP, elevated HuR further increases cyclin E1, a key positive regulator for G1/S transition, and promotes mitosis [82, 90]. Furthermore, CIRP accelerates G0/G1 and G1/S transitions by inhibiting the phosphorylation of cyclin D1 and p27 via the kinase Dyrk1b/Mirk [91]. In addition, CIRP appears to promote cell cycle progression from S phase to G2/M phase [92]. Hence, the current understanding is that CIRP facilitates cell proliferation.

*Apoptosis*: Apoptosis is induced by numerous exogenous and endogenous signals, involves various signaling pathways, and occurs in a broad range of diseases [93]. RNA-binding proteins are largely known as a family of proteins that modulate apoptosis [94]. Many studies have revealed that CIRP mediates, at least partially, the hypothermic protection of cells from apoptosis. Specifically, CIRP suppresses apoptosis in neural stem cells [95] and cortical neurons probably through mitochondrial pathways [96], which might mediate the protective effect of therapeutic hypothermia [97]. The inhibition of p53, Fas, and caspase-3 pathways also contributes to CIRP-mediated anti-apoptotic effects [57, 98, 99].

*Senescence*: CIRP activates the ERK1/2 pathway by increasing the phosphorylation of ERK1/2, which promotes cell division and bypasses replicative senescence [92, 100, 101]. The activation of ERK1/2 by CIRP contributes to tumor growth in pituitary corticotroph adenoma [102]. In addition, CIRP has been found at the telomere of HeLa cells [103]. A recent study has unraveled a novel role of CIRP in the maintenance of telomerase activity in both normothermic and hypothermic conditions [104]. These studies collectively support a role of CIRP in anti-senescence.

### Biological functions and diseases

#### Brain disorders

Therapeutic hypothermia can not only efficiently reduce primary injury and prevent secondary injury in acute ischemia [7] and spinal cord injury (SCI) [105], but also delay the progression of chronic neurodegenerative diseases [106]. In vitro, the two cold-inducible proteins CIRP and RBM3 both function against apoptosis in cultured primary neurons or neuron-like PC12 cells [34, 96, 107].

The role of CIRP in brain ischemic injury is controversial. *CIRP* mRNA levels measured by Northern blot were found to decrease 3–6 h after transient ischemia in rat hippocampus but remained unchanged in the cerebral cortex during a 48 h observation period [56]. However, in the same ischemic model, real-time RT-PCR showed a gradual increase in *CIRP* mRNA by approximately fivefold until 24 h after cerebral ischemia in rat cortex [108]. In contrast to ischemia, hypothermia considerably induced CIRP expression by approximately 30-fold until 24 h, and the combination of hypothermia and ischemia did not further enhance the CIRP level from 30-fold [108].

An elevated level of ROS is one important detrimental factor in the induction of oxidative stress during ischemia–reperfusion injury in the brain [109]. In PC12 cells, CIRP expression has been observed to be downregulated upon H_2_O_2_ treatment, which produces ROS [56]. When CIRP is induced endogenously or overexpressed artificially, H_2_O_2_-induced apoptosis in cultured neural cells is dramatically inhibited, indicating a neuroprotective role of CIRP [57, 92]. In contrast to this beneficial intracellular action of CIRP, release of CIRP into the blood system is associated with the activation of detrimental immune responses. Zhou et al. reported that the secretion of CIRP from microglia after cerebral ischemia with subsequent CIRP-mediated TNF-α expression leads to neuroinflammation and causes neuronal damage both in vivo and in vitro [110]. An investigation of alcohol-induced brain inflammation has also demonstrated that extracellular CIRP mediates neuroinflammation by upregulating TNF-α and IL-1β [111]. To summarize, CIRP exerts opposing functions during brain ischemia–reperfusion injury. On the one hand, as long as CIRP remains intracellularly localized, it protects neurons from apoptosis; on the other hand, once CIRP is released, e.g., from microglia, it mediates devastating neuroinflammation at the cellular level.

#### Circadian rhythm

CIRP and RBM3 show high homology with the two glycine-rich RNA-binding proteins, AtGRP7, and AtGRP8, in *Arabidopsis thaliana* (Fig. 1). Both of these proteins are pivotal components in a circadian-regulated feedback loop [24, 112]. Similarly, CIRP expression is diurnally regulated in the suprachiasmatic nucleus (SCN) and cerebral cortex of mice [113]. The expression level of CIRP peaks at 6 pm and reaches the bottom at 3 am, altering in a light-dependent manner; the fluctuation occurs only in juvenile and adult mice but not in neonates [113]. Similar to mammals, light signal has been observed to induce CIRP expression in amphibian brain [114]. In 2012, CIRP was identified as a regulator of circadian oscillator genes, including the *CLOCK* gene, in a post-transcriptional pattern in mammals [71]. Further experiments showed that CIRP is upregulated during the sleep phase [115].

In mammals, the central clock in the SCN systematically synchronizes the body temperature cycles to environmental light–dark cycles [116] and to peripheral clocks (e.g., the liver and pancreas) [117, 118]. A recent study has shown that CIRP contributes to temperature-sensitive oscillation in murine hepatocytes, linking the subtle fluctuation in mammalian body temperature and circadian rhythm in peripheral tissues [118]. Furthermore, as the liver and pancreas are essential metabolic organs, nutrition necessarily influences the peripheral clock in these organs [119]. Ketogenic diets and fasting, which disrupt peripheral clocks, induce CIRP expression in the liver [120]. Meal timing greatly enhances the circadian expression of CIRP in pancreatic adenocarcinoma, pointing to a link between circadian rhythm and cancer therapy involving CIRP [121]. Therefore, CIRP is believed to be a component of mammalian circadian oscillation, which not only is regulated by body temperature and responds to changes in the environment, such as light in a subtle manner, but also controls the expression of downstream circadian genes.

#### Immune response

Conserved from plant to mammals, pattern recognition receptors (PRRs) are primitive key components to identify pathogen-associated molecular patterns (PAMP) and damage-associated molecular patterns (DAMP) [122, 123]. In 2007, the plant cold-inducible protein, AtGRP7, was first discovered to be involved in plant immunity [25]. AtGRP7 significantly enhances PAMP-triggered immunity by binding to the transcripts and proteins of two PRRs, FLS2 and EFR [124]. It is believed that the mammalian cold-inducible proteins are also involved in innate immune response. In 2013, CIRP was identified as a novel inflammatory mediator released from the heart and liver into the circulatory system during hemorrhagic shock and sepsis [125]. Secreted CIRP acts as a DAMP by binding to TLR4–MD2 complex (one class of mammalian PRRs), triggering inflammatory response by stimulating TNFα and HMGB1 secretion [125]. Conversely, CIRP expression can be impaired by TNFα or TGFβ [126], suggesting a negative feedback loop. Similar to the dual role of CIRP in the brain as discussed above, a recent report has shown that hypothermia-induced CIRP expression in the liver protects hepatocytes by reducing ROS production [127], while anti-CIRP antibody treatment, which neutralizes secreted CIRP in the serum, significantly decreases the inflammatory response and protects the liver from ischemic-reperfusion injury [128]. Other anti-CIRP therapies are also considered to treat inflammation-related disorders, such as abdominal aortic aneurysm based on animal experiments [129]. Today, the quantitative measurement of CIRP levels in peripheral blood using an ELISA kit appears achievable, opening the possibility to investigate CIRP as a new diagnostic marker for sepsis [130]. Furthermore, CIRP deficiency accelerates inflammation phase and wound healing process [131]. Overall, extracellular CIRP induces cell damage by inducing inflammatory responses. However, in the late stage of inflammation, the damaged cells are eliminated by inflammation, and regenerated cells can substitute the dysfunctional ones [132]. It implies that CIRP-mediated immune response may also have favorable aspects.

#### Cancer

Deduced from the above-summarized features, CIRP and RBM3 are both involved in cell cycle regulation and cell proliferation and are both present in proliferating and malignant cells. Therefore, they are considered as proto-oncogenes, promoting cancer cell proliferation, and transformation in vitro [36, 133, 134] and are differentially expressed in a variety of different cancers compared with normal tissues (Table 2). Despite these common features, their roles in clinical cancer development seem to be opposite. RBM3 expression always correlates with good prognosis and reduced risk of disease progression and recurrence, whereas CIRP seems to be an indicator of poor prognosis (Table 2). Accordingly, high CIRP level is associated with poor chemosensitivity of cancer cells [135, 136].

**Table 2:** Roles of CIRP and RBM3 in cancer

Cancer type	CIRP or RBM3 studied				Proposed mechanisms	Prognosis associated with high expression	References
Breast cancer	Both		RBM3		–	Good	[181]
			CIRP		Increase cyclin E1	Poor	[82, 142]
Epithelial ovarian cancer	RBM3				Inhibit MCM3, Chk1 and Chk2	Good	[171, 182]
Endometrial carcinoma	CIRP				–	Unclear	[141]
Prostate cancer	RBM3				Involve ERG and PTEN; enhance chemo-sensitivity; regulate CD44 splicing	Good and unclear	[135, 163, 183–185]
Testicular non-seminomatous germ cell cancer	RBM3				–	Good	[187]
Urothelial bladder cancer	RBM3				–	Good or not determined	[188, 189]
Oropharyngeal squamous cell carcinoma	Both			RBM3	–	Not determined	[193]
				CIRP	Induce TLR4-related inflammation	Poor	[140]
Esophageal and gastric adenocarcinoma	RBM3				–	Good	[194]
Liver cancer	CIRP				Increase ROS, IL-1β and IL-6; suppress p53	Poor	[99, 137]
Colorectal cancer	Both	RBM3			Suppress GSK3β activity and enhance β-catenin signaling	Good	[169, 190–192]
		CIRP			Induce TNF-α and IL-23	Poor	[138]
Melanoma	RBM3				Inhibit MCM3	Good	[195–197]
Astrocytoma	RBM3				–	Unclear	[198]
Pituitary adenoma	CIRP				Induce cyclin D1 and decrease p27 via Erk1/2 signaling	Poor	[102, 139]

CIRP is differentially expressed in many cancer types [101]. In hepatocellular carcinoma (HCC), CIRP has been proposed to promote carcinogenesis by controlling ROS accumulation and cancer stem/progenitor cell expansion, and risk of HCC recurrence is positively correlated with Cirp expression in liver [137]. In colorectal tumors, CIRP links tumorigenesis and chronic inflammation by stimulating cytokines, including TNF-α and IL-23 [138]. Thus, inhibiting CIRP could be of therapeutic value at least in liver cancer treatment [99].

In pituitary adenoma, high CIRP expression correlates with proliferating invasive and recurrent tumor, probably via ERK1/2 signaling pathway [102, 139]. In oral squamous cell carcinoma, CIRP is co-expressed with TLR4 and is associated with a short survival rate [140]. In endometrial carcinoma and some endometrial hyperplasia, CIRP expression is absent or markedly decreased compared with normal endometrium, indicating a role of CIRP in normal proliferative events [141]. In addition, CIRP is thought to be involved in the progression of ductal carcinoma into invasive breast cancer presumably by increasing the expression of CyclinE1, an important cell cycle regulator, thereby promoting proliferation and tumor progression in breast cancer transformation [82, 90, 142].

In summary, increasing numbers of clinical studies have demonstrated that CIRP is linked to poor clinical outcome in cancer. Among a variety of possible mechanisms, we would like to highlight the following consideration based on the available above-described data. Major inflammatory pathways are involved in carcinogenesis [143]. The extracellular action of CIRP in immune responses has been linked to detrimental consequences [125]. Albeit no data have yet been published regarding similar extracellular action in cancer, it is hypothesized that extracellular CIRP signaling in cancers may contribute to tumor progression and worse outcome through cytokine activation, as supported by the works from Sakurai et al. [137, 138].

#### Reproduction

As reported above, two decades ago CIRP was found in the germ cells of mammalian testis [40]. The working temperature of testis is physiologically lower than the core body temperature, which is believed to favor spermatogenesis as well as CIRP expression. Several mechanisms have been proposed to explain the temperature-sensitive function of CIRP in spermatogenesis and testicular injury protection. Post-transcriptionally, CIRP binds and stabilizes mRNAs relating to male infertility in testis [72]. Experimentally increased scrotal temperature causes in testis and epididymis a reduced expression of CIRP and an increase in germ cell apoptosis [144]. In addition, CIRP reduces cryptorchidism-induced testicular damage by suppressing pro-apoptotic p53 and Fas [98]. Upon testicular torsion/detorsion, CIRP prevents testicular damages probably by decreasing oxidative stress and apoptosis in germ cells [145]. Furthermore, CIRP regulates key pathways or components in the cell cycle that affect spermatogenic functions. A comprehensive study has revealed that CIRP promotes the proliferation of undifferentiated spermatogonia by interacting with Dryk1b/Mirk [91]. In addition, when CIRP is downregulated, p44/p42, p38, and SAPK/JNK MAPK pathways are activated in germ cells and impair spermatogenesis [146]. In turtle, CIRP is involved in sex determination in a temperature-dependent manner [147] but with unclear results in the American alligator [148].

Oocytes and embryo cryopreservation are of great importance in reproductive medicine [149]. To freeze an egg or embryo, a novel flash-freeze process called vitrification has replaced the traditional slow-cooling method with more benefits [149]. During vitrification of oocytes, some genes, including CIRP, change their expression profiles and are believed to be involved in the protection against cold stress or crystallization [150, 151]. However, the involvement of CIRP in embryo cryopreservation is controversial. One study has demonstrated that CIRP is elevated in post-warming pro-nuclear stage embryos with higher expression levels in 8-cell stage embryos in the vitrification group [152]. A second group reported that no significant difference in CIRP expression was observed between vitrification and slow-freezing in 8-cell stage embryos [153]. CIRP is also associated with the enhancement of developmental competence of vitrified-warmed oocytes [66]. In addition, high CIRP expression level has been found in cryopreservation of reproductive organs, such as calf testis and sheep ovary [154, 155].

## Regulation and functions of RBM3

### Molecular regulation of RBM3

There is little information on the mechanisms regulating RBM3 expression. In alternative splicing, a long *RBM3* isoform, annotated only in mouse, is more abundant in sleep deprived mice as compared with the commonly known *RBM3* transcript in mammals and *CIRP*, which are both under-expressed during sleep deprivation, suggesting that *RBM3* isoforms may play different roles in circadian oscillation in mice [156]. In mammalian neurons, an *RBM3* splicing variant lacking a single arginine shows a higher dendritic localization, compared with the isoform having this specific arginine, which may be due to arginine methylation [48] (see paragraph ‘spatial distribution’).

In 2001, Chappell SA et al. discovered an IRES in the 5′-leader sequence of *RBM3* [157, 158], which has been applied in viral studies or as a biotechnological tool until very recently [159–161]. However, the presumed RBM3 IRES was unmasked as a cloning artifact in which a cDNA for the *Thoc1* gene on chromosome 18 had recombined with an *Rbm3* cDNA [162].

### Molecular and cellular activities of RBM3

#### Regulation of post-transcriptional and translational events

RBM3 can bind to and alter the translation of mRNA, as shown for *COX*-*2*, *IL*-*8*, and *vascular endothelial growth factor* (*VEGF*) in macrophages or cancer cells [65, 133], presumably in a cell type-specific manner [36] and involving the interaction with HuR [133]. In addition, RBM3 associates with the spliceosome and is involved in splicing [79]. In prostate cancer cells, RBM3 represses the variant *v8*–*v10* of *CD44* mRNA to inhibit stemness and tumorigenesis but enhances the standard spliced *CD44* transcript [163]. In addition, RBM3 can modulate alternative polyadenylation in the same way as CIRP [78, 164].

RBM3 is known to modulate the translational process in several ways. In general, RBM3 enhances global protein translation [48, 165]. The underlying mechanisms of this enhancement include (1) binding to 60S ribosomal subunits in an RNA-independent manner; (2) increasing the formation of active polysomes; (3) dephosphorylating eukaryotic initiation factor 2 alpha (eIF2α); and (4) facilitating the phosphorylation of eukaryotic initiation factor 4E (eIF4E) [48, 165]. However, whether RBM3 can regulate translation via microRNAs remains controversial (see below). Moreover, our recent study revealed many ribosomal proteins associating with RBM3 [47], supporting its role in promoting translation. Whether this involvement of RBM3 in translational processes distinguishes fundamentally RBM3 from CIRP is unclear yet as CIRP has not been well-studied in translational processes.

#### Regulation of microRNA biogenesis

MicoRNAs (miRNAs) are small non-coding RNA molecules with approximately 21 nucleotides. These molecules are widely found in different organisms and regulate numerous developmental and cellular processes post-transcriptionally. Most miRNAs are generated in vivo from the canonical pathway: transcribed primary precursors (pri-miRNAs) are processed by the Drosha/DGCR8 complex into 70-nucleotide precursors (pre-miRNAs) in the nucleus; pre-miRNAs are further exported into cytoplasm and processed by Dicer complex to generate mature miRNA duplexes. One strand of the duplex is incorporated into RISC to guide the degradation of target mRNA [166]. RBM3 is considered to alter miRNA levels, thereby contributing to global protein translation, e.g., under hypothermia [165]. RBM3 binds to 70-nucleotide precursors and facilitates their processing by Dicer complex [167]. RBM3 appears to positively modulate the majority of miRNAs, and negatively modulate only a minor group of miRNAs. However, the finding that RBM3 upregulates most miRNAs is contradictory to the fact that RBM3 increases overall translation. In particular, mature miR-125b expression decreases when RBM3 is overexpressed, as shown by Dresios et al., but the opposite was demonstrated by Pilotte et al., making the RBM3-mediated modulation of specific miRNAs controversial [165, 167]. In addition, a recent study has demonstrated that reduced RBM3 level promotes the expression of a small subset of temperature-sensitive miRNAs, which targets immune genes and prevents pathological hyperthermia [168]. Therefore, RBM3 is believed to execute a regulatory function in miRNA expression, although its exact role remains largely unclear.

#### Signaling pathways

*Stemness*: Similar to CIRP, RBM3 is involved in the Wnt/β-catenin signaling pathway as well. In colorectal cancer cells, RBM3 induces stemness through a mechanism involving suppression of GSK3β kinase activity, thereby enhancing β-catenin signaling [169].

*Cell cycle*: Similar to CIRP, recent studies confirmed a positive role of RBM3 in promoting cell cycle progression. RBM3 modulates cell cycle in G2/M transition [133, 170], instead of G0/G1 and G1/S transitions for CIRP [91]. Specifically, knockdown of RBM3 in tumor cells increases caspase-mediated apoptosis coupled with nuclear cyclin B1, and phosphorylated Cdc25c, Chk1, and Chk2 kinases, implying that under conditions of RBM3 downregulation, cells undergo mitotic catastrophe [133]. Mouse embryonic fibroblasts from RBM3-deficient mice show markedly increased number of G2-phase cells [170], confirming that RBM3 is essential for cells to progress through mitosis. This may explain why tumors with high RBM3 expression show increased sensitivity to chemotherapy and, thus, are associated with better prognosis as compared with RBM3 low or even negative tumors [171].

*Apoptosis*: RBM3 inhibits staurosporine-induced apoptosis in neuron-like PC12 cells by repressing PARP cleavage [34]. The induction of Bcl-2 and suppression of caspase expression may also be involved in RBM3-mediated survival [47, 172].

*ER stress*: In the presence of ER stress, unfolded proteins accumulate in the ER lumen and activate unfolded protein response (UPR) to rescue cells. If ER stress exists continuously, UPR initiates the apoptotic program. PERK-eIF2α-CHOP signaling is one of the three main branches of UPR, and it plays the most important role in UPR-induced apoptosis [173]. Under sustained ER stress, RBM3 represses the phosphorylation of PERK and eIF2α, which leads to a decrease in CHOP expression and rescue cells from UPR-induced apoptosis [47]. Notably, although RBM3 is induced by hypothermia, hypothermia itself can activate UPR without inducing apoptosis [42]. Upon ischemia-induced prolonged ER stress, hypothermia has a protective effect by suppressing UPR to prevent apoptosis [174], in accordance with Zhu et al.’s report [47].

### Biological functions and diseases

#### Brain disorders

Very recently, Peretti et al. revealed an important neuroprotective role of RBM3 in Alzheimer’s and prion disease models [175]. Notably, RBM3, but not CIRP, is significantly induced by hypothermia in mouse, preventing neuronal loss and restoring synapse reassembly [175]. Although the underlying mechanism is unknown, one hypothesis may involve the suppression of eIF2α kinase PERK, which has been shown to be a potential therapeutic target in Alzheimer’s disease-related deficits of synapse plasticity [176]. The activity of PERK has recently been linked to RBM3 [47]. However, the details of this mechanism remain to be elucidated.

In response to acute injuries to brain or spinal cord, RBM3 instantly changes its temporal and spatial distributions similar to CIRP. After spinal cord injury (SCI) in a rat model, the number of RBM3-positive cells increases with varying temporal dynamics reported [177, 178]. In one report, RBM3 expression peaked at 1 day after SCI [178], while in the other, RBM3 significantly increased 1 day post-SCI but did not reach the maximal expression level until 5 days post-SCI [177]. The spatial expression of RBM3 in these two reports is also inconsistent. Zhao et al. reported that mostly primary neurons and only a few astrocytes were positive for RBM3 expression under normal conditions, and that RBM3 was induced in both neurons and astrocytes [178]. In the other study, RBM3 is present in both neurons and astrocytes in the sham group, and only astrocytic RBM3 could respond to SCI-induced stress [177]. This discrepancy may result from the instable surgical conditions, although both reports generally support the hypothesis that RBM3 is inducible upon SCI and may exert important pathophysiological functions.

Until now, studies have supported the notion that RBM3 is a general neuroprotective effector, while CIRP can either protect neuronal cells or induce massive neuronal death by mediating neuroinflammation once released.

#### Circadian rhythm

RBM3 has been studied less frequently in circadian rhythm than CIRP. As described above, RBM3 modulates circadian oscillation by controlling alternative polyadenylation in cooperation with CIRP [78]. In patients suffering from neurological diseases with dysregulation of the sleep–wake cycle, such as bipolar disorders and cluster headache, RBM3 was the most significantly altered gene in peripheral lymphoblasts [179]. In mice, two alternatively spliced RBM3 isoforms may play different roles in sleep [156].

#### Immune response

In RBM3 knockout mice, no obvious change in cytokine expression has been found in a model of DNA-mediated innate immune response compared with wild-type [170]. Very recently, reduced RBM3 levels have been found to associate with reduced expression of some immune genes, such as *IL*-*6* and *TLR2*, by modulating a subset of thermos-miRNAs [168]. To the best of our knowledge, no data published thus far suggest that RBM3 might be actively released as it has been demonstrated for CIRP [125].

#### Cancer

A large variety of immunohistochemical studies, including many tumor types, have shown consistently that loss of RBM3 expression is associated with clinically more aggressive tumors and an independent factor of poor prognosis.

Breast cancer is the leading cancer type in women, and RBM3 is overexpressed in this cancer [180], with a direct correlation of RBM3 expression level and improved clinical outcome [181]. In female genital organs, RBM3 correlates with favorable cisplatin sensitivity and good prognosis in epithelial ovarian cancer (EOC) [182], presumably via a mechanism by which RBM3 suppresses the poor prognostic markers MCM3, Chk1, and Chk2, which are all involved in DNA integrity and cell cycle [171].

In males, prostate cancer is one of the most common cancer types. Very similar to breast cancer and EOC, a high level of RBM3, as an independent biomarker in prostate cancer, predicts a low risk of disease progression and recurrence [183]. Interestingly, high RBM3 expression is found in poorly differentiated prostate tumor [184], whereas experimental downregulation of RBM3 in prostate cancer cells attenuates cell survival and enhances chemosensitivity in vitro [135]. These observations are consistent with the role of RBM3 in promoting cell proliferation and survival but cannot explain the favorable prognosis, indicating the involvement of other mechanisms. One study suggests that the activation of ERG and the depletion of PTEN may contribute to RBM3-mediated good prognosis in radically operated prostate cancer [185]. Another study indicates that RBM3 attenuates the stemness and tumorigenesis of prostate cancer cells by inhibiting CD44 variant splicing [163], although this finding is in contrast to findings in colorectal cancer [169]. Specific cancer types likely differentially affect RBM3 signaling as is known for other proteins, such as estrogen receptors [186]. Moreover, low RBM3 in testicular non-seminomatous germ cell cancer correlates with high risk of treatment failure [187]. Reduced RBM3 levels in urothelial bladder cancer are associated with tumor progression and poor prognosis [188], while high RBM3 expression correlates with lower stage tumors and decreased risk of lymphovascular invasion [189].

Colorectal cancer is the most common type of cancer of the digestive system, and a high level of RBM3 expression is associated with improved prognosis [190], whereas a loss of RBM3 expression is associated with poor prognosis and right-sided localization [191]. Therefore, RBM3 has been proposed as a potential prognostic biomarker, especially in young patients [192].

In the upper digestive system, RBM3 is downregulated in HPV-negative oropharyngeal squamous cell carcinoma compared with normal oral mucosa [193]. In esophageal and gastric adenocarcinoma, high nuclear expression of RBM3 is beneficial, and it correlates with intestinal metaplasia-associated tumors and predicts low risk of recurrence and death independently [194].

Furthermore, low RBM3 expression in other cancer types is associated with poor survival as well. RBM3 is expressed in malignant melanoma [195], and low expression of RBM3 is associated with tumor progression and poor prognosis [196]. High MCM3 expression is observed with a reduced RBM3 level similar to epithelial ovarian cancer [197]. The only exception published thus far is astrocytoma, where higher expression of RBM3 is associated with a higher grade and may promote astrocytic carcinogenesis [198].

A pivotal feature of most tumors is hypoxia [199]. The hypoxic cancer stem cell niche provides a microenvironment for the maintenance of immature cancer cells [200], and moderates hypoxia triggers the induction of RBM3 [54]. In fact, experiments in colorectal cancers have shown that RBM3 regulates Wnt/β-catenin signaling to induce the stemness of cancer cells [169], in contrast to findings in prostate cancer cells [163]. However, the clinical data gathered on RBM3 in various tumor types (Table 2) with RBM3 as a marker of better outcome point toward a more complex network of RBM3 interaction than considered so far. Various specific cell types likely affect RBM3 baseline expression and functions differentially; the cell environment also has an important impact on RBM3.

In conclusion, whereas both CIRP and RBM3 show common characteristics of proto-oncogenes at the cellular level, their roles in the clinical tumor setting are diverse, with CIRP as a marker of poor prognosis and RBM3 as a marker of good prognosis.

#### Reproduction

Distinct from CIRP, RBM3 is predominantly expressed in Sertoli cells of mammalian testis, rather than in germ cells [41]. Sertoli cells are “nurse” cells to nourish germ cells in the process of spermatogenesis, indicating that RBM3 may only play a supportive role, which is different from a direct role of CIRP. In male rats, the expression level of RBM3 in the sexually dimorphic nuclei of the preoptic area (SDN-POA) neurons is almost twofold as that in female rats, although it declines by 50 % upon NMDA receptor inactivation. In contrast, the expression level of RBM3 in females is unaffected by an NMDA receptor inhibitor, indicating a dose-dependent mechanism related to X-chromosome inactivation [201].

Similar to CIRP, RBM3 is also involved in the vitrification of oocytes [202] and embryos [152, 153], as well as in the cryopreservation of genital organs [154], indicating overlapping activities of RBM3 and CIRP in cold-mediated cell and tissue protection.

#### Other functions

As RBM3 is involved in a variety of transcriptional and translational events, it is not surprising that it also exerts functions in viral infection. Together with hnRNP A2, RBM3 can interact with vaccinia viral proteins and contribute to the late transcription of virus [203, 204]. More details remain to be determined for RBM3-mediated viral infection and a potential relation to immune response.

In a model of muscle atrophy, rats with hindlimb suspension show higher expression levels of RBM3 in soleus muscles [205]. In particular, RBM3 is involved in the regulation of skeletal muscle size and the prevention of muscle loss, indicating a novel vital function of RBM3 in muscle disease [205]. A subsequent study has further revealed that RBM3 inhibits both necrosis and apoptosis in muscle myoblasts, consistent with a general cytoprotective function of RBM3 [172]. Moreover, RBM3 is suggested to mediate hypothermia-induced overexpression of bone protein alkaline phosphatase and osteocalcin [206]. These studies open new avenues to the understanding of multiple functions of RBM3.

## Conclusion and outlook

**Fig. 2:**
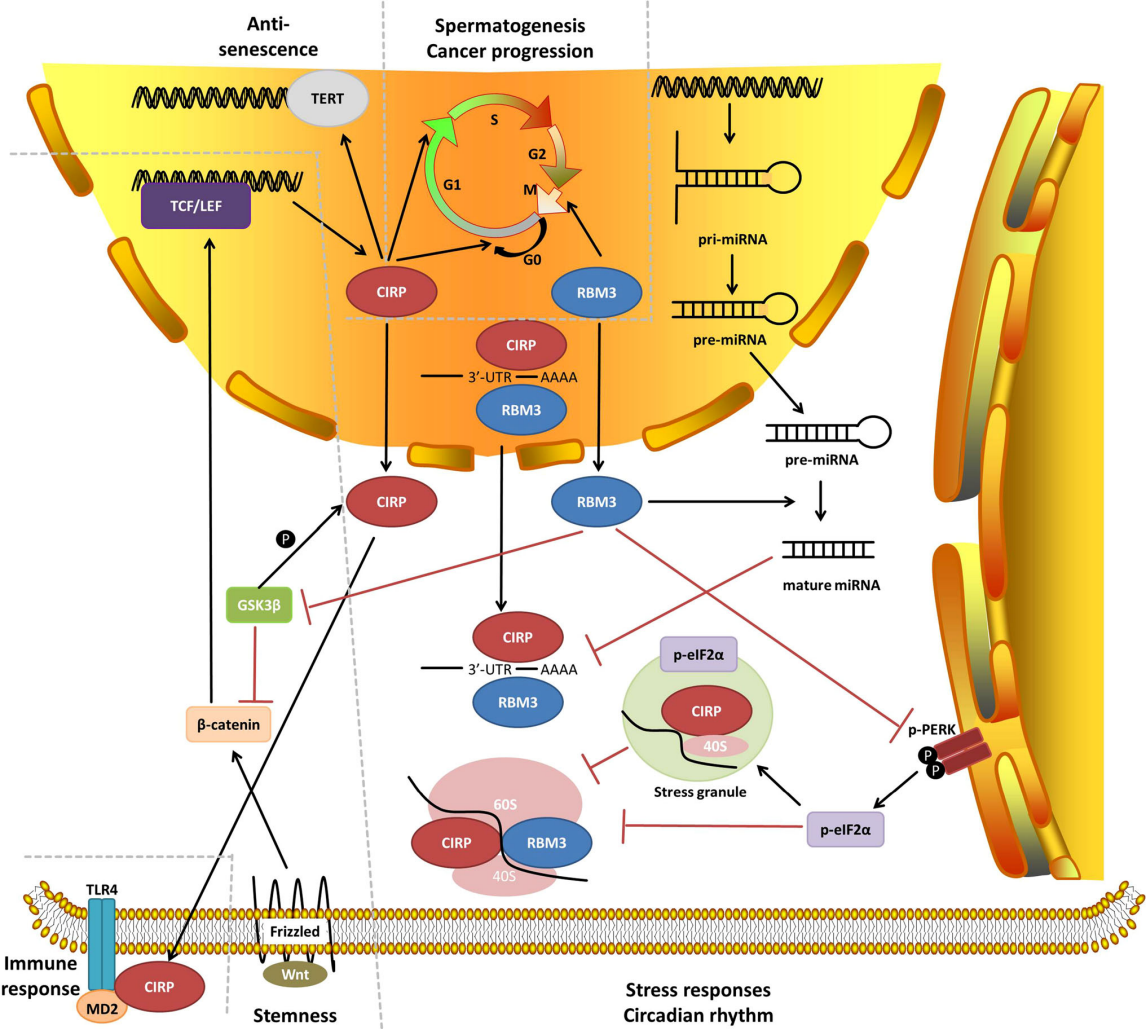
Molecular network of CIRP and RBM3 functions. Key molecular and cellular functions are briefly illustrated, and their relationships to physiological and pathological functions are indicated

In this review, we comprehensively summarized the biological activities of CIRP and RBM3 by elucidating the upstream and downstream molecular and cellular aspects, and highlighting their relationships to various physiological and pathological processes in vivo (Fig. 2).

When studying the various research papers on CIRP and RBM3, a particularly striking observation was the variety of diverse cellular functions in which CIRP and RBM3 are involved. In contrast, the upstream mechanisms regulating CIRP and RBM3 appear to be rather similar, including cellular stressors, such as hypothermia and hypoxia. CIRP and RBM3 are unique cellular tools that are present in many cell types that can be activated by various cellular stressors and that are applied by the cells depending on the specific cellular context, namely, the specific presence of other stress response molecules.

Despite the many similarities between both CIRP and RBM3 proteins, particularly regarding evolutionary conservation, sequence homology, expression, and inducibility, their biological functions are distinct. Whereas both proteins are generally upregulated in cancer tissues compared with normal tissue, RBM3 has been identified unanimously as a biomarker for favorable outcome; CIRP for poor outcome. One reason might be the fact that RBM3 facilitates mitosis and increases chemo sensitivity of tumor cells. A second reason for this disparity in cancer progression might be related to the ability of secretion. CIRP has been identified as an important mediator upon severe inflammation or ischemia with specific detrimental functions aggravating cell damage, whereas RBM3 has not been identified extracellularly thus far.

Future research on CIRP and RBM3 in mammals will benefit from reviewing the findings made so far in plants on their homologues AtGRP7 and AtGRP8. And a key for therapeutic implications of CIRP and RBM3 in the future is the ability to translate the current knowledge on the effects of CIRP and RBM3 into specific therapeutic approaches that target either the two proteins directly or the signaling pathways in which they are involved.
